# A Study on the Fracture Properties of Cement Asphalt Emulsion Mixture Based on the SCB Test

**DOI:** 10.3390/ma18091967

**Published:** 2025-04-25

**Authors:** Yunliang Li, Qichen Wang, Xu Li, Yue Zhao, Wenyang Yu, Baocheng Liu, Yiqiu Tan

**Affiliations:** School of Transportation Science and Engineering, Harbin Institute of Technology, Harbin 150090, China

**Keywords:** cement asphalt emulsion mixture, SCB test, peak load, fracture energy, Mode I fracture

## Abstract

Cement asphalt emulsion mixture (CAEM) is a composite material composed of asphalt emulsion, cement, and graded aggregates. Currently, CAEM is primarily applied as a base course material for highways to improve the cracking resistance of pavement structures. To achieve this goal, the fracture performance of CAEM plays a crucial role. Experimental studies have demonstrated that the fracture behavior of CAEM exhibits a significant correlation with the amount of asphalt emulsion and binder used. The influence of asphalt emulsion and binder content on the fracture parameters of CAEM was investigated through semi-circular bending (SCB) tests, combined with analyses of peak load and fracture energy. Furthermore, the influences of temperature, loading rate, and notch depth on fracture performance were evaluated. The microstructure of the cured binder was characterized by scanning electron microscopy (SEM), while the deformation behavior of CAEM was assessed through creep tests. The experimental results indicate that, to ensure satisfactory fracture resistance in CAEM, the optimal content of asphalt emulsion should be controlled within the range of 2.0~3.0%, with a corresponding binder content of 6.0%. This study provides theoretical and practical guidance for the material design optimization of CAEM, with a specific focus on enhancing fracture resistance performance.

## 1. Introduction

Cement asphalt emulsion mixture (CAEM) is composed of cement, asphalt emulsion, and graded aggregates [1,2,3,4]. Using asphalt emulsion and cement as binders, CAEM can be mixed and constructed at ambient temperature, offering simplified construction procedures and enhanced field applicability [5,6,7,8]. Compared to conventional asphalt mixtures and semi-rigid base materials, CAEM demonstrates higher strength while maintaining high flexibility. Based on the material characteristics of CAEM, its primary application is currently in highway base courses to mitigate cracking issues prevalent in conventional semi-rigid base pavements [9]. The fracture performance of CAEM is critically important, as it is closely related to the cracking resistance of asphalt pavements [10]. Consequently, systematic research into the fracture behavior of CAEM is imperative to align with its engineering objectives as a crack-resistant pavement material.

The factors influencing the fracture performance of CAEM are complex. CAEM, as an organic–inorganic composite material, is composed of cement, asphalt, and aggregates with significantly different material properties. In particular, as CAEM utilizes both cement and asphalt as composite binders, its material properties are more complex than those of either asphalt or cement alone [11,12]. The presence of asphalt imparts distinct viscoelastic characteristics to the mechanical behavior of the composite binder [13], while the microstructure of the cured binder remains relatively complex [14]. The specific material composition and microstructural features make the mechanical properties of the composite binder more susceptible to environmental influences [15]. Compared with the composite binder, the structural composition of CAEM is even more complex, comprising cement hydration products, asphalt films, and aggregates, as well as voids and free water encapsulated by asphalt films [16]. Therefore, CAEM exhibits complexity in both material and structural compositions [17,18,19], making the study of its fracture performance challenging [20,21,22]. The key factors affecting the fracture performance of CAEM include asphalt emulsion and binder content, temperature, and loading rate. Among the factors influencing the fracture performance of CAEM, the content of asphalt emulsion and binder directly affects the material composition and structural configuration of CAEM, exhibiting significant correlations with its fracture properties. In addition to material composition factors, fracture testing conditions such as temperature and loading rate also influence the fracture performance of CAEM [23,24,25,26].

The selection of appropriate testing methods is critical for experimental investigations into the fracture properties of materials. In studies on the fracture performance of asphalt mixtures and cement concrete, researchers have employed various methods, including the SCB test, the indirect tensile (IDT) test [27,28], the disk-shaped compact tension (DCT) test [29,30], the notched beam bending (NBB) test [31,32], the edge-notched disc bend (ENDB) test [33,34], and the Arcan test. These methods enable the determination of fracture parameters such as peak fracture load, fracture energy, fracture work, fracture toughness, and fracture displacement, which are used to characterize the fracture behavior of materials. Among these experimental methods, SCB testing provides clearer fracture mode visualization and a more intuitive analysis of fracture mechanisms, making it the selected methodology for investigating CAEM’s fracture performance in this study.

Current research on the fracture performance of CAEM remains limited in scope, whereas cement asphalt mortar (CAM), composed of cement, asphalt emulsion, and sand as a composite binder system, has been the subject of extensive investigation. Comprehensive studies on CAM’s fracture behavior were conducted by Shima Najjar et al. [35,36,37,38], covering fracture modes, fracture parameters, influencing factors, and material design criteria. Based on these investigations, an optimized mix design method for CAM was developed. The effects of temperature, loading rate, and other factors on the fracture performance of CAM were elucidated, and the influence of asphalt aging was also examined. Fereidoon Moghadas Nejad et al. [39,40] focused on the effects of the mix’s design variables and loading rate, demonstrating their quantifiable impact on the fracture performance of CAM. The stress intensity factor of CAM was analyzed by Wang Chao et al. [41] through load–deflection curve analysis. Xie Yongjiang et al. [42] analyzed the temperature-dependent fracture toughness of CAM. These investigations collectively highlight the methodological framework applicable to CAEM fracture studies. The asphalt content is the primary factor influencing the fracture performance of CAM, as the presence of asphalt imparts a certain degree of temperature sensitivity to its fracture behavior. Considering the viscoelastic nature of CAM, Song Zhao et al. [43] investigated its fracture performance based on viscoelastic fracture theory, analyzing the viscoelastic fracture parameters of CAM and comparing them with those derived from linear elastic and elastic–plastic fracture models. To enhance the fracture performance and durability of CAM, Amir Hossein Saesaei [44] incorporated additives such as silica fume, metakaolin, and granite sludge, which effectively improved its resistance to acid attack. In addition, the incorporation of fly ash and slag has also been shown to significantly improve the material properties of CAM, particularly in enhancing its long-term strength development [45].

This study investigated the fracture performance of CAEM using the SCB test, focusing on the effects of asphalt emulsion and binder content. The influences of temperature, loading rate, and notch depth on fracture behavior were also analyzed. The experimental results revealed the influencing mechanisms of asphalt emulsion content, binder content, temperature, loading rate, and notch depth on the fracture performance of CAEM, with optimal ranges determined for emulsion content and binder content. Overall, this research provides instructive guidance for enhancing the fracture resistance of CAEM in engineering applications.

## 2. Material and Mix Design

### 2.1. Asphalt Emulsion and Cement

The primary materials of CAEM include P.O.42.5 ordinary Portland cement, cationic medium-setting asphalt emulsion, and aggregates. In accordance with the testing standards specified in JTJ 052-2000 and JTG E03-2005 [46,47], the material properties of asphalt emulsion and cement were tested, as shown in Table 1 and Table 2.

### 2.2. Aggregates and Gradation

The technical properties of coarse aggregates, fine aggregates, and mineral filler were tested by the relevant testing standards [48]. Their technical properties are presented in Table 3, Table 4 and Table 5, respectively.

**Figure 1:**
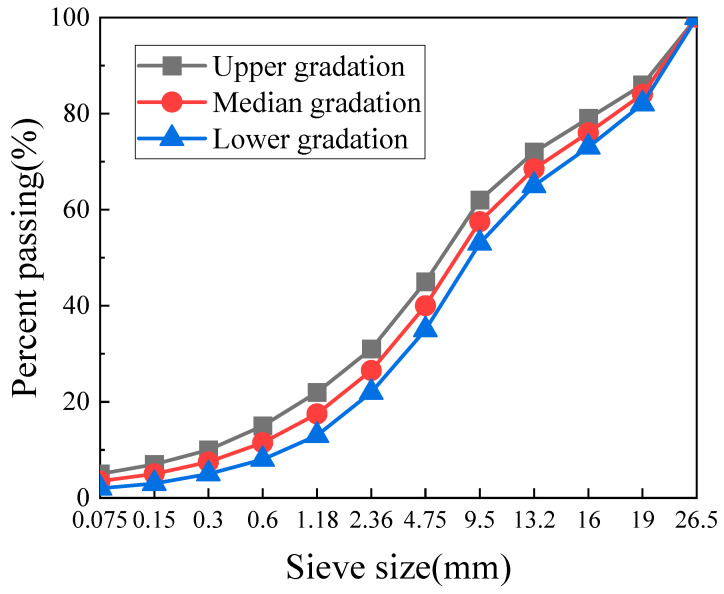
Gradation curve of CAEM.

CAEM is applied in the asphalt pavement’s base course layer using the gradation of AC-25. According to the relevant standard [49], the median gradation of AC-25 was adopted as the target gradation for CAEM, which is illustrated in Figure 1.

### 2.3. Asphalt Emulsion and Binder Content

CAEM employs cement and asphalt emulsion as composite binders, where the contents of these binding materials influence the fracture characteristics [50]. In current studies on CAEM, the asphalt emulsion content (AEC) typically ranges from 1.5% to 3.5%, while the cement content (CC) generally falls within the range of 2.0% to 3.5% [51,52]. Based on the existing literature, this research selected five levels of asphalt emulsion content and four levels of cement content. The total binder content (BC) was controlled within the range of 4.0% to 7.0%, and the cement-to-asphalt emulsion ratio (CC/AEC) was maintained between 0.57 and 2.33. A total of 17 different CAEM mix designs were prepared, with the water-to-binder ratio fixed at 0.9. The mix proportions of the 17 specimens are presented in Table 6.

## 3. Specimen Preparation and Experimental Program

### 3.1. Specimen Preparation

During the preparation of CAEM, an asphalt mixing machine was employed to ensure the homogeneous dispersion of cement and asphalt emulsion. According to the mix proportions specified in Table 6, the required amounts of asphalt emulsion, cement, and water were measured. Aggregates of each size fraction were weighed based on the grading curve, and all aggregates were thoroughly dried. The mixing procedure followed these sequential steps: water was first blended with the asphalt emulsion for 1 min, followed by adding cement with continued mixing for 3 min. Subsequently, aggregates and mineral filler were incorporated into the mixture and mixed for 3 min to complete the mixing process. All mixing operations were conducted at ambient temperature.

Cylindrical specimens with a diameter and height of 15 cm were fabricated using the vibratory compaction method. The mixed material was placed into the mold in three layers. The mold was then positioned on the platform of the vibration compaction machine, and the vibrating compaction head was inserted into the mold. Under vibration, the mixture was compacted, while the change in specimen height was continuously monitored during the compaction process. Vibration was stopped once the specimen reached the target height, marking the completion of compaction. After demolding the specimens 48 h post-compaction, they were transferred to a curing chamber maintained at 20 °C and 65% relative humidity for 28 days of standard curing.

**Figure 2:**
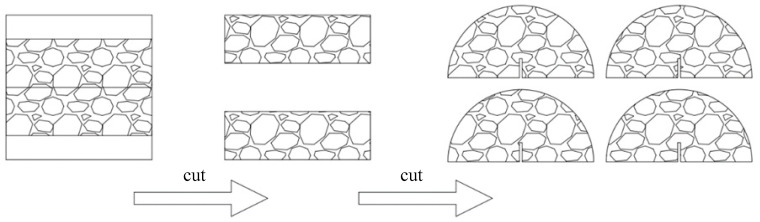
Fabrication of semi-circular specimens.

Following the curing period, the cylindrical specimens were sectioned by removing uneven portions at both ends to obtain four semi-circular specimens with a uniform thickness of 5 cm. Prefabricated notches were then cut to the specimens with controlled notch depths (ND) of ND_1_ = 10 mm, ND_2_ = 20 mm, and ND_3_ = 30 mm. The notch preparation process is illustrated in Figure 2.

### 3.2. SCB Experimental Testing Program

**Figure 3:**
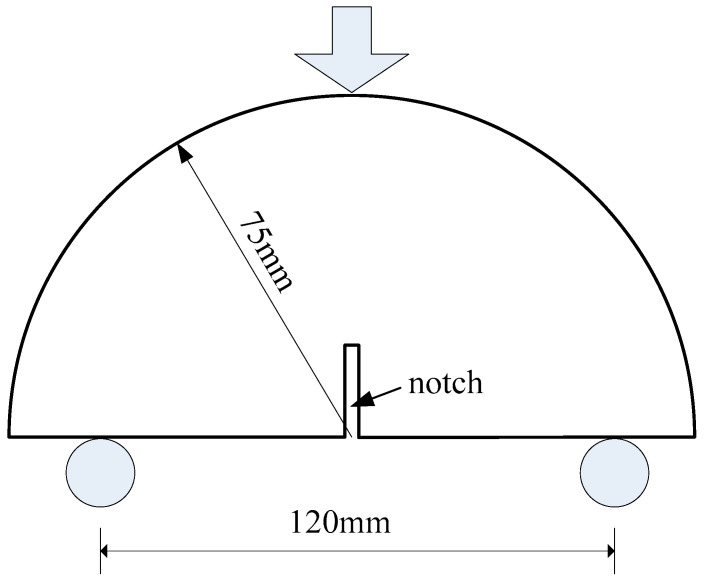
Geometry of specimens.

The fracture performance of CAEM was assessed using the SCB test performed on a universal test machine (UTM) with a support span of 120 mm, which is shown in Figure 3. Testing variables included three temperature levels (T_1_ = 20 °C, T_2_ = 0 °C, and T_3_ = −20 °C), three loading rates (LS_1_ = 1 mm/min, LS_2_ = 2 mm/min, and LS_3_ = 3 mm/min), and three notch depths (ND_1_ = 10 mm, ND_2_ = 20 mm, and ND_3_ = 30 mm). All combinations of these parameters are listed in Table 7.

### 3.3. Fracture Parameters

**Figure 4:**
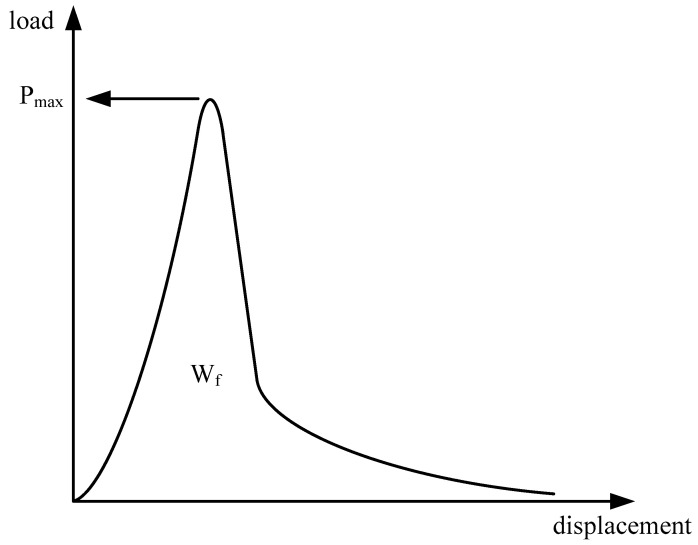
Calculation of fracture parameters.

The peak load (*P_max_*) can be obtained from load–displacement curves, as shown in Figure 4, and the fracture energy (*G_f_*) of the specimen can be calculated by the following equations:

(1) $$G_{f}=\frac{W_{f}}{A_{lig}}$$

(2) $$A_{lig}=(r-D)\times B$$

where *W_f_* represents the fracture work, defined as the area under the load–displacement curve obtained through integration, *A_lig_* is the net area of the fracture zone, *r* is the specimen radius, *D* is the notch depth, and *B* indicates the specimen thickness.

### 3.4. Microstructural Test

The binder in CAEM consists of cement and asphalt emulsion. Upon mixing cement with asphalt emulsion, the hydration of cement and the breaking process of asphalt emulsion start to occur, leading to the solidification of the cement–asphalt emulsion composite binder and subsequent strength development. Variations in asphalt emulsion content alter the microstructural characteristics of the cured binder, thereby influencing the fracture performance of CAEM. Binder specimens with cement-to-asphalt emulsion ratios of 1.75, 1.20, 0.83, and 0.57 were prepared. These specimens were cured for 28 days under controlled conditions of 20 °C and 65% relative humidity. Scanning electron microscopy (SEM) was then used to examine the microstructure of the cured binder, in order to analyze the influence of asphalt emulsion content on its microstructural characteristics.

### 3.5. Creep Test

A creep test was adopted to investigate the deformation capacity of CAEM, which is closely related to its fracture performance. Marshall specimens with cement-to-asphalt emulsion ratios of 1.75, 1.20, 0.83, and 0.57 were prepared and cured for 28 days under controlled conditions (20 °C, 65% relative humidity). Uniaxial compressive creep tests were then conducted using UTM at a constant temperature of 20 °C, with the creep stress set to 10% of the compressive strength.

## 4. Analysis and Discussion

### 4.1. Fracture Process Analysis

**Figure 5:**
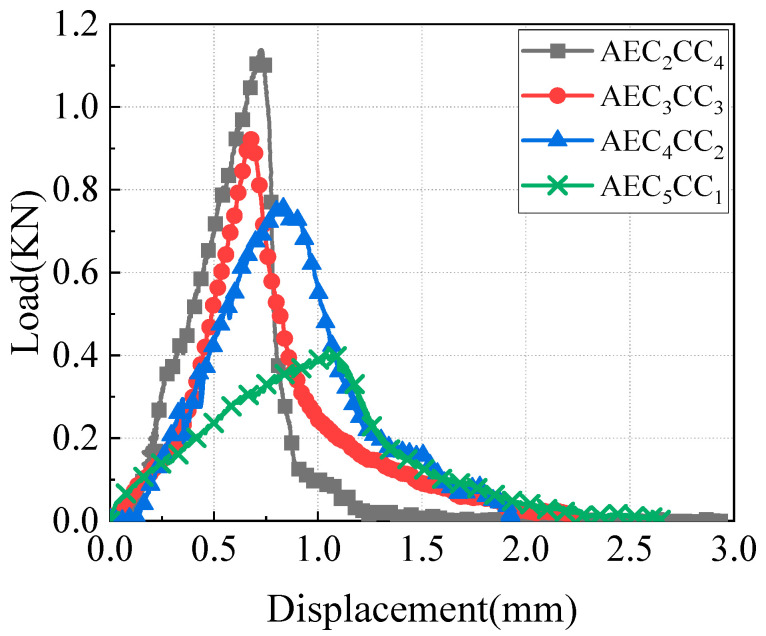
Fracture mode of CAEM (test condition T_1_LS_1_ND_3_: T_1_ = 20 °C, LS_1_ = 1 mm/min, ND_3_ = 30 mm).

The load–displacement curves of specimens AEC_2_CC_4_, AEC_3_CC_3_, AEC_4_CC_2_, and AEC_5_CC_1_ with a binder content of 5.5% are shown in Figure 5. These four specimens had cement-to-asphalt emulsion ratios of 1.75, 1.20, 0.83, and 0.57, with asphalt emulsion contents of 2.0%, 2.5%, 3.0%, and 3.5%, respectively. With the increase in asphalt emulsion content, both the rising and falling slopes of the load–displacement curve decreased, the peak load was reduced, and the deformation at failure increased. The characteristics of the load–displacement curves from CAEM fracture tests demonstrate that CAEM underwent brittle fracture at lower asphalt emulsion contents and ductile fracture at higher asphalt emulsion contents. The asphalt emulsion content had a pronounced influence on the fracture mode of CAEM. As the asphalt emulsion content increased, the deformation capacity of CAEM was enhanced, allowing for greater deformation at fracture and exhibiting ductile fracture behavior.

### 4.2. Influencing Factors of Fracture Parameters

#### 4.2.1. Asphalt Emulsion Content

The binder in CAEM combines two materials with fundamentally different characteristics: cement and asphalt. Incorporating asphalt into a cement-based binder is intended to enhance flexibility and deformability, thereby improving crack resistance. However, CAEM’s crack resistance depends on both strength and deformation capacity. Cement-based binders, such as those used in semi-rigid base materials, exhibit high strength but low deformation capacity, resulting in brittle fracture under stress. In contrast, asphalt-based binders, like those in asphalt mixtures, offer superior deformation capacity but insufficient strength for structural applications. Therefore, the cracking resistance of pavement materials can be optimized by achieving an optimal balance between sufficient strength and superior deformation capacity, thereby satisfying the structural and functional requirements of the base course layer.

**Figure 6:**
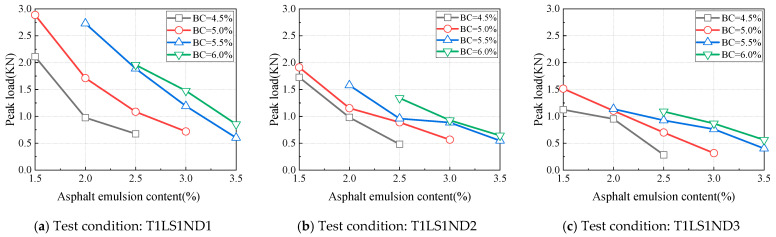
Influence of asphalt emulsion content on peak load.

Including asphalt in CAEM’s binder influences the material’s strength and, consequently, its peak load. Under different test conditions, with binder contents set at 4.5%, 5.0%, 5.5%, and 6.0%, the variations in peak load during the specimen fracture process were investigated relative to changes in asphalt emulsion content, and the results are shown in Figure 6. When the binder content was held constant, the peak load decreased as the asphalt emulsion content increased. The relationship between peak load and asphalt emulsion content is approximately linear. Consequently, relying solely on peak load is insufficient to comprehensively evaluate the impact of asphalt emulsion content on the fracture performance of CAEM.

**Figure 7:**
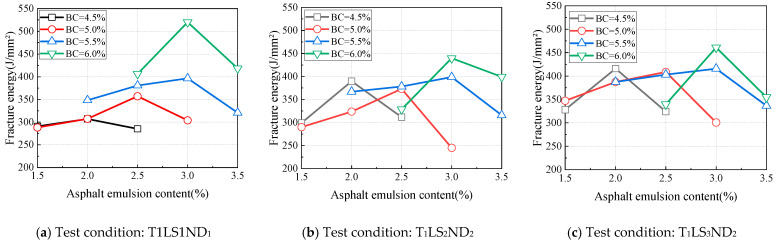
Influence of asphalt emulsion content on fracture energy (20 °C).

The fracture energy (*G_f_*) was calculated using Equation (1). Under different testing conditions, with binder contents fixed at 4.5%, 5.0%, 5.5%, and 6.0%, the variations in fracture energy with asphalt emulsion content were determined, and the results are illustrated in Figure 7. As the asphalt emulsion content increased, the fracture energy initially increased and then decreased, indicating the existence of a maximum fracture energy at an optimal asphalt emulsion content. For binder contents of 4.5%, 5.0%, 5.5%, and 6.0%, the asphalt emulsion contents corresponding to the maximum fracture energy were 2.0%, 2.5%, 3.0%, and 3.0%, respectively. As the binder content increased, the asphalt emulsion content required to achieve the maximum fracture energy also increased, with the optimal asphalt emulsion content for maximizing fracture energy falling within the range of 2.0~3.0%. Furthermore, when the binder content exceeded 5.5%, the asphalt emulsion content corresponding to the maximum fracture energy remained constant.

**Figure 8:**
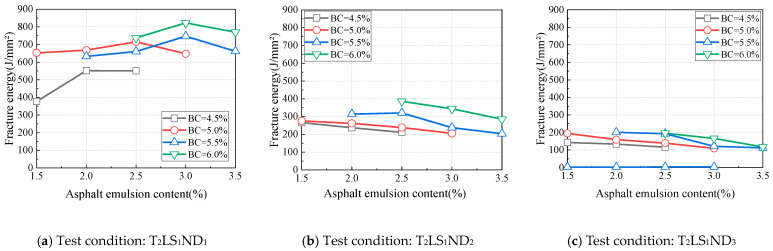
Influence of asphalt emulsion content on fracture energy (0 °C).

**Figure 9:**
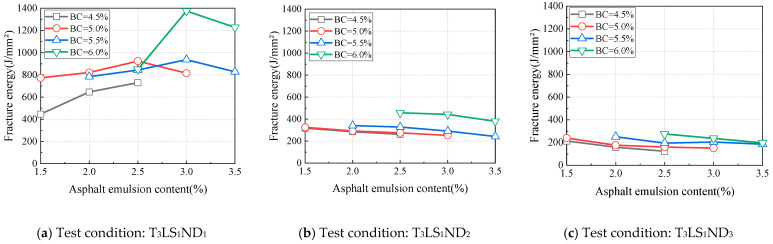
Influence of asphalt emulsion content on fracture energy (−20 °C).

Under test temperatures of 0 °C and −20 °C, the variations in fracture energy with asphalt emulsion content under different test conditions were analyzed, and the results are shown in Figure 8 and Figure 9, respectively. The relationship between fracture energy and asphalt emulsion content critically depended on notch depth at these subzero temperatures. For a notch depth of 10 mm, the fracture energy variation pattern remained consistent with the 20 °C test condition (Figure 8a and Figure 9a). However, fracture energy exhibited a monotonic decrease at 20 mm and 30 mm notch depths with an increase in the asphalt emulsion content (Figure 8b,c and Figure 9b,c), contrary to the observation at 20 °C.

Fracture energy is a parameter derived from elastic–plastic fracture mechanics and primarily correlates with the material’s plastic deformation capacity. As defined by Equation (1), fracture energy is proportional to the fracture work under a given notch depth. The fracture work is calculated as the area under the load–displacement curve. Accordingly, the fracture work depends on the peak load and the post-peak curve area, which is governed by the material’s deformability. Therefore, the fracture energy of CAEM reached its maximum value, and its crack resistance attained optimal performance when the material achieved an optimal balance between strength and deformation capacity. As evidenced by the preceding analysis, the peak load consistently decreased with an increase in asphalt emulsion content under all test conditions, indicating strength reduction in CAEM. Conversely, the deformation capacity of CAEM improved with higher asphalt emulsion content. At 20 °C, the strength of CAEM decreased, while its deformability increased with an increase in the asphalt emulsion content; thus, the fracture energy of CAEM attained its peak value at a specific amount of asphalt emulsion content.

CAEM exhibited reduced ductility under the test conditions of 0 °C and −20 °C, where the net area of the fracture zone of the specimen had a pronounced influence on its deformation capacity. For specimens with a shallow notch depth (10 mm), the net area of the fracture zone was larger, resulting in a longer crack propagation path during fracture. These specimens also exhibited greater deformation at complete fracture. Therefore, the fracture energy varied with asphalt emulsion content following the same trend as that observed at 20 °C, and a maximum value of fracture energy was observed with changes in asphalt emulsion content. For specimens with greater notch depths (20 mm and 30 mm), the net area of the fracture zone was smaller, resulting in shorter crack propagation paths during fracture. Upon complete failure, reduced deformation was observed, and the influence of deformation capacity on fracture energy diminished. In these cases, fracture energy was primarily governed by the peak load of the specimen. As established in the prior analysis, peak load decreased with an increase in asphalt emulsion content. Consequently, specimens with deeper notch depths exhibited a decreasing trend in fracture energy under low-temperature conditions as asphalt emulsion content increased.

#### 4.2.2. Influence of Binder Content on Fracture Parameters

In the binder of CAEM, increasing cement content enhances the CAEM’s strength. According to the inherent properties of asphalt, materials utilizing asphalt as the primary binder typically exhibit an optimal asphalt content at which peak strength is achieved, as observed in asphalt mixtures. Excessive asphalt content compromises material strength. The crack resistance of CAEM depends on material strength and deformation capacity. While cement and asphalt emulsion content can affect the strength of CAEM, the deformation capacity of CAEM is primarily controlled by asphalt emulsion content. Therefore, the binder content significantly influences the fracture performance of CAEM. As a critical parameter in CAEM mix design, systematic investigation of binder content effects on fracture behavior is imperative.

**Figure 10:**
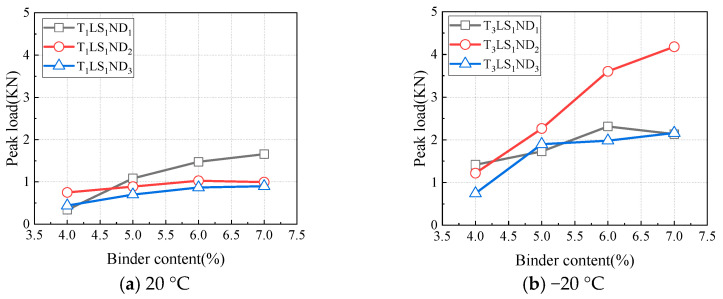
Influence of binder content on peak load.

The test results of specimens AEC_2_CC_1_, AEC_3_CC_2_, AEC_4_CC_3_, and AED_5_CC_4_ were analyzed, with these four specimens having a cement-to-asphalt emulsion ratio of 1.0 and binder contents of 4.0%, 5.0%, 6.0%, and 7.0%, respectively. As shown in Figure 10, the peak load varied with binder content under different test conditions. Under all test conditions, the peak load exhibited a similar trend. When the binder content was below 6.0%, the peak load increased with higher binder content; once the binder content exceeded 6.0%, the increasing trend of peak load slowed down, or the peak load began decreasing, which aligns with the strength variation observed in asphalt mixtures influenced by asphalt content. Therefore, controlling the binder content within 6.0% is reasonable from the perspective of peak load.

**Figure 11:**
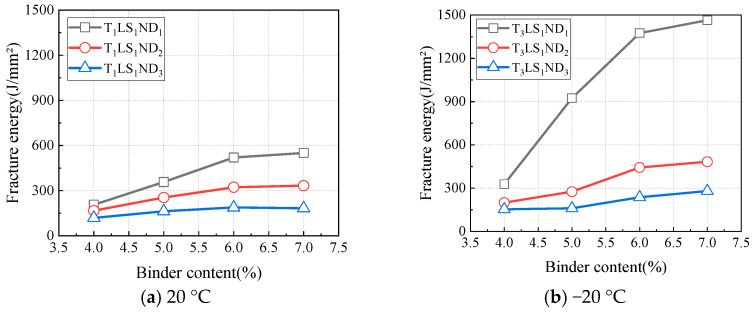
Influence of binder content on fracture energy.

The variations in fracture energy with binder content for specimens AEC_2_CC_1_, AEC_3_CC_2_, AEC_4_CC_3_, and AED_5_CC_4_ under different test conditions are shown in Figure 11. The fracture energy exhibited a two-stage trend with increasing binder content. It increased continuously when the binder content ranged from 4.0% to 6.0%, while the increasing trend slowed once the binder content exceeded 6.0%. This behavior is consistent with the previously observed variation pattern of peak load with binder content. Based on the trends of both peak load and fracture energy with binder content variation, controlling the binder content within 6.0% is reasonable for CAEM design, ensuring enhanced crack resistance.

#### 4.2.3. Influence of Temperature on Fracture Parameters

**Figure 12:**
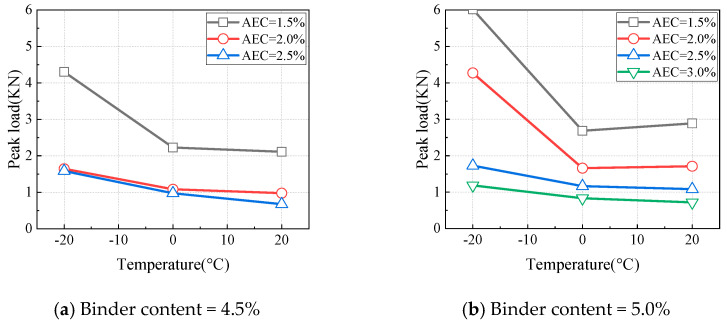

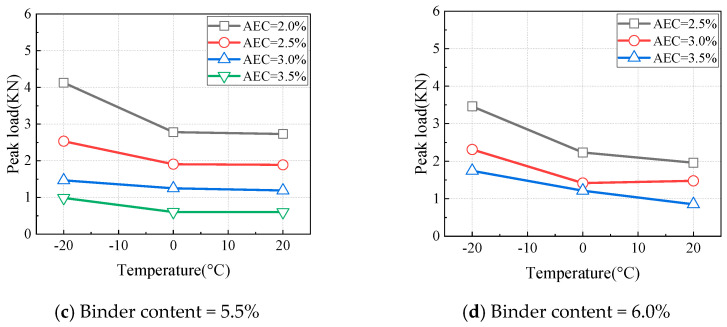
Influence of temperature on peak load.

Since asphalt is a temperature-sensitive material, the fracture performance of CAEM likewise exhibited temperature sensitivity. The variations in peak load with temperature under test conditions of a loading rate of 1 mm/min, a notch depth of 10 mm, and temperatures of 20 °C, 0 °C, and −20 °C for different binder contents are shown in Figure 12. As shown in the figure, the peak load of CAEM decreased with an increase in temperature. This reduction occurs because the asphalt within CAEM softens at elevated temperatures, leading to decreased material strength and a corresponding decline in peak load.

**Figure 13:**
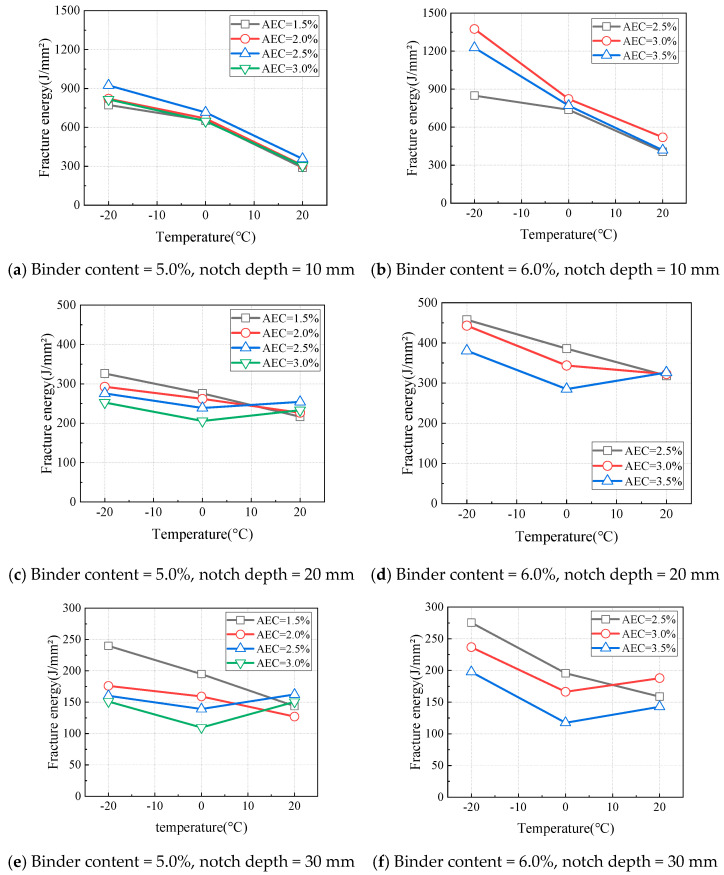
Influence of temperature on fracture energy.

Figure 13 illustrates the variations in the fracture energy of CAEM with test temperature under different testing conditions. When the notch depth was 10 mm (Figure 13a,b), the fracture energy decreased as the temperature increased. When the notch depth was 20 mm or 30 mm (Figure 13c–f), the relationship between fracture energy and temperature depended on the asphalt emulsion content. Specifically, when the asphalt emulsion content was less than 2.0%, the fracture energy decreased with an increase in temperature. However, when the asphalt emulsion content exceeded 2.0%, the fracture energy first decreased and then increased as the temperature increased. The fracture performance of CAEM was determined by both the peak load and the deformation capacity. The peak load was primarily influenced by the asphalt emulsion content and temperature, while the deformation capacity depended on the asphalt emulsion content, temperature, and notch depth.

When the notch depth was 10 mm, the net area of the fracture zone was larger, and the crack propagation path was longer. Under these conditions, CAEM exhibited good deformation capacity across different asphalt emulsion contents. Consequently, the fracture energy was mainly governed by the peak load at this notch depth. As the temperature increased, the peak load decreased, leading to a corresponding reduction in fracture energy.

When the notch depth was 20 mm or 30 mm, the variation in fracture energy with temperature depended on the asphalt emulsion content. When the asphalt emulsion content was less than 2.0%, the fracture energy decreased as the temperature increased. This is primarily because, at low asphalt emulsion contents, the specimens exhibited brittle fracture behavior, and the fracture energy was mainly governed by the peak load. As the temperature increased, the peak load decreased, resulting in a corresponding reduction in fracture energy.

At notch depths of 20 mm and 30 mm, with asphalt emulsion content greater than 2.0%, the fracture energy showed a trend of initially decreasing and then increasing with rising temperature. This indicates that, at higher asphalt emulsion contents, the deformation capacity of CAEM becomes more sensitive to temperature variations. At temperatures of −20 °C and 0 °C, due to the relatively limited deformation capacity of CAEM, the fracture energy was predominantly influenced by the peak load. As the temperature increased, the peak load decreased, leading to a decline in fracture energy when the temperature increased from −20 °C to 0 °C. At a temperature of 20 °C, the asphalt softened, enhancing the deformation capacity of CAEM. The positive effect of temperature on improving deformation capacity outweighs the negative impact of the reduced net area of the fracture zone. As a result, the overall deformation capacity of CAEM increased with rising temperature. Under these conditions, the fracture energy was primarily determined by the deformation capacity of CAEM. Therefore, as the temperature increased from 0 °C to 20 °C, the fracture energy of CAEM increased.

Based on the previous findings, the optimal asphalt emulsion content is recommended to be in the range of 2.0% to 3.0%. The above analysis further indicates that the low-temperature crack resistance of CAEM is mainly governed by the peak load, while the high-temperature crack resistance is primarily controlled by the deformation capacity.

#### 4.2.4. Influence of Notch Depth (ND) on Fracture Parameters

**Figure 14:**
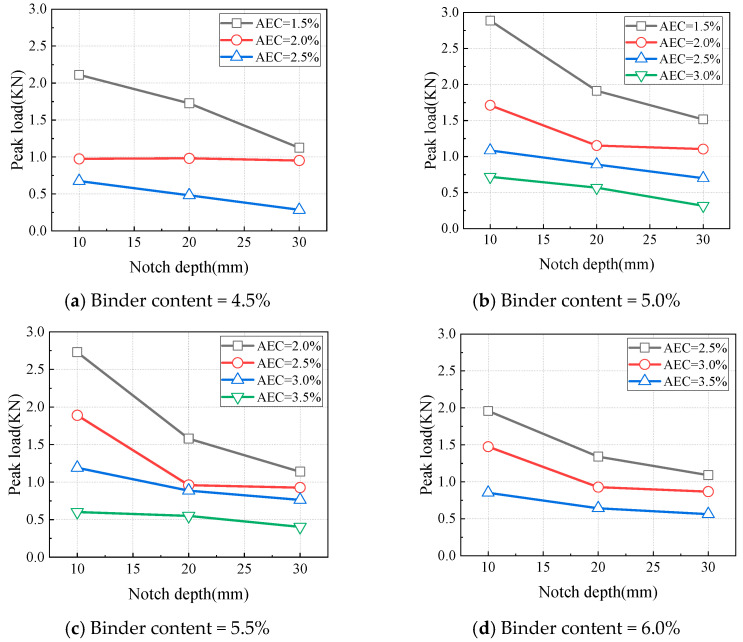
Influence of notch depth on peak load (T = 0 °C, LR = 1 mm/min).

The prefabricated notch in the SCB specimen ensures Mode I fracture initiation, and the notch depth characterizes the influence of initial material defects on crack resistance. At a temperature of 0 °C and 1 mm/min loading rate, the variations in CAEM’s peak load with notch depth are shown in Figure 14. The peak load of CAEM decreased with increasing notch depth. A deeper notch depth reduced the net area of the fracture zone, thereby lowering the specimen’s resistance to crack propagation.

**Figure 15:**
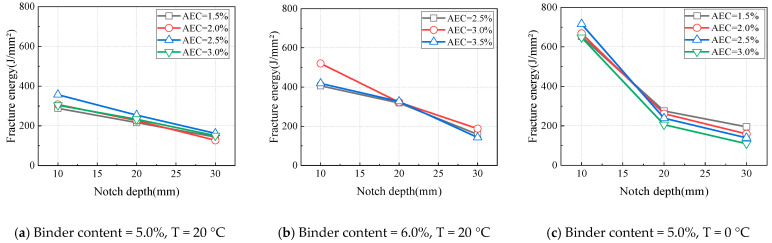

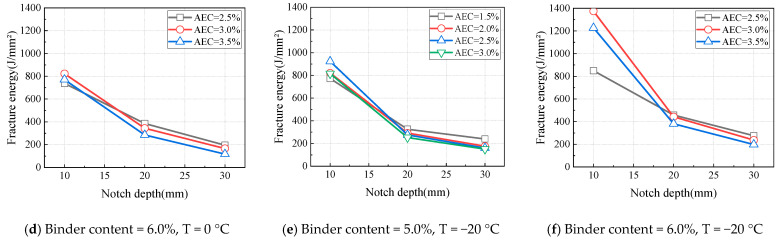
Influence of notch depth on fracture energy (LR = 1 mm/min).

At a loading rate of 1 mm/min, the variations in the fracture energy of CAEM with notch depth under different temperature conditions are shown in Figure 15. At all test temperatures, the fracture energy of CAEM decreased with an increase in notch depth. As analyzed previously, the peak load decreased with deeper notches. Simultaneously, increased notch depth reduced the net area of the fracture zone and shortened the crack propagation path, thereby limiting deformation during fracture. Since both specimens’ peak load and deformation capacity decreased with an increase in notch depth, the fracture energy consequently declined as notch depth increased.

#### 4.2.5. Influence of Loading Rate (LR) on Fracture Parameters

**Figure 16:**
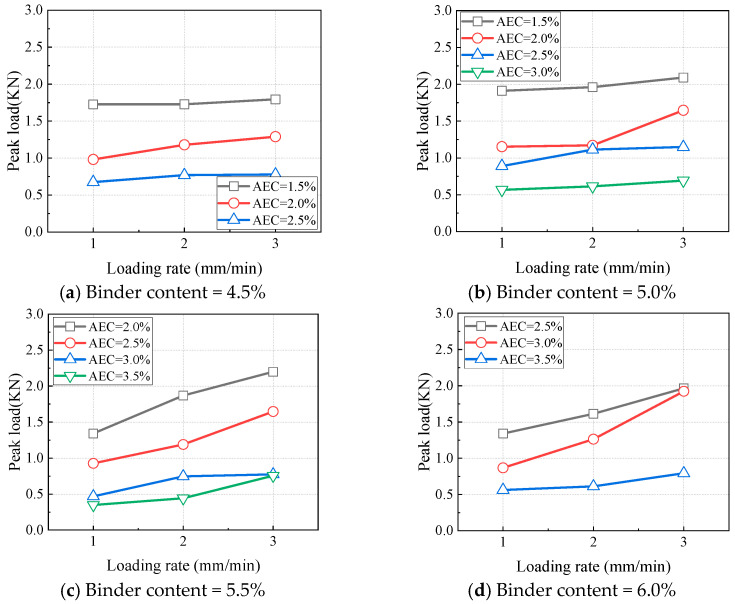
Influence of loading rate on peak load (T = 0 °C, ND = 20 mm).

**Figure 17:**
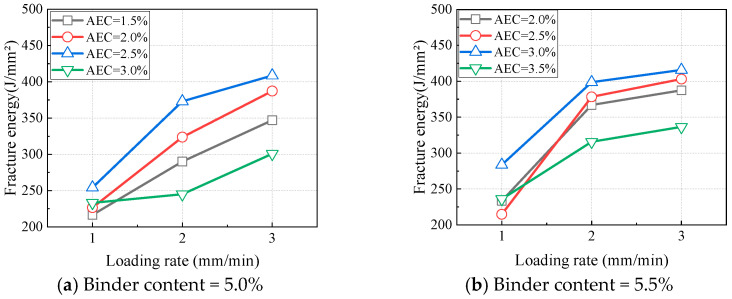
Influence of loading rate on fracture energy (T = 0 °C, ND = 20 mm).

Due to the presence of asphalt, CAEM is classified as a viscoelastic material. In addition to temperature, the loading rate also influences the peak load. At 0 °C with a notch depth of 20 mm, the variations in peak load with the loading rate are illustrated in Figure 16. Under all the tested material proportions, the peak load increased with an increase in the loading rate. Similarly, the variations in the fracture energy of CAEM with the loading rate are shown in Figure 17, according to which the fracture energy also increased with higher loading rates. Based on the viscoelastic properties of asphalt, under high loading rate conditions, asphalt exhibited mechanical behavior analogous to that observed at low temperatures. As the viscosity of asphalt increased, its strength was enhanced. This elevated viscosity improved the overall strength of CAEM, thereby causing the peak load of CAEM to increase with higher loading rates. The augmented peak load further enhanced the fracture energy of CAEM.

### 4.3. Sensitivity Analysis of Influencing Factors

**Figure 18:**
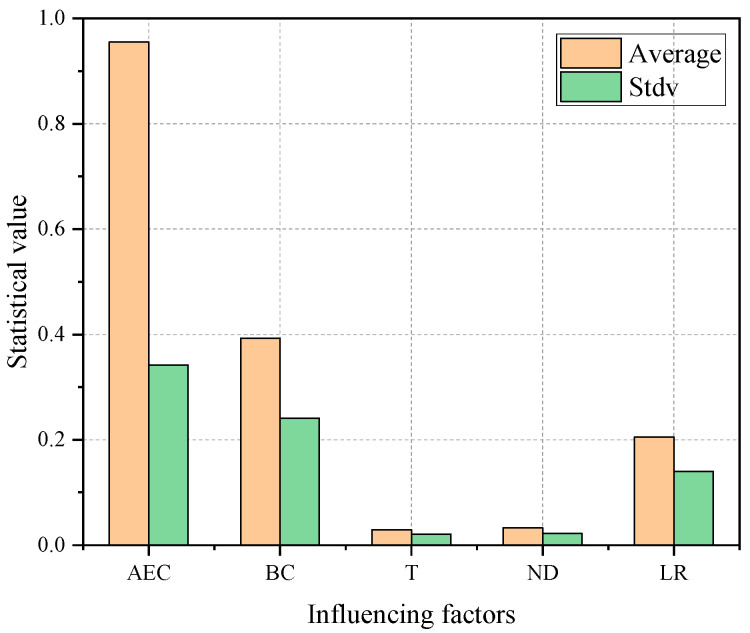
Mean values and standard deviations of influencing factors.

The effects of asphalt emulsion content, binder content, temperature, notch depth, and loading rate on the fracture parameters of CAEM were analyzed individually. To further compare the correlations and sensitivities of these factors with respect to the fracture parameters, a statistical analysis was performed. The preceding results indicated that the peak load exhibited an approximately linear relationship with each of these influencing factors. Accordingly, linear regression was conducted between the peak load and each factor, and the slopes of the regression equations were obtained. The absolute value of the slope serves as an indicator of the sensitivity of the peak load to each factor, with larger absolute values indicating a greater influence on the peak load. The mean values and standard deviations of the absolute slopes for each influencing factor, obtained through linear fitting under different conditions, are presented in Figure 18.

Based on the average slopes, the order of influence of the five factors on the fracture performance of CAEM is as follows: asphalt emulsion content, binder content, loading rate, notch depth, and temperature. This indicates that the fracture performance of CAEM is primarily governed by material composition, with the asphalt emulsion content and binder content being the most critical factors. The effect of the loading rate is related to the viscoelastic mechanical properties of CAEM, while changes in notch depth alter the net area of the fracture zone, thereby exerting a significant impact on the peak load. Among these five factors, temperature has the least influence, which further demonstrates that the incorporation of cement reduces the temperature sensitivity of CAEM’s fracture performance. Moreover, the relatively large standard deviations of the fitted slopes suggest notable variability among the different influencing factors. This is mainly because the fitted data are derived from average values under varying test conditions, where interactions and coupling effects among the factors contribute to data dispersion. Therefore, further research is recommended for a more in-depth exploration of the interrelationships and combined effects of these factors.

### 4.4. Microstructural Characterization of Cured Binder Composite

**Figure 19:**
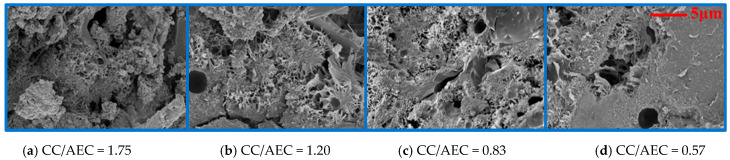
Microstructure of CAEM.

CAEM utilizes cement and asphalt emulsion as the binder. Upon mixing, the hydration process of cement and the breaking process of asphalt emulsion initiate simultaneously. Cement generates hydration products through hydration reactions, while asphalt emulsion undergoes a breaking process, leading to the separation of asphalt from water and the formation of continuous asphalt films. These asphalt films intertwine with cement hydration products, collectively forming the binder. The cement-to-asphalt emulsion ratio governs the material composition of hydration products versus asphalt films in the binder, which in turn determines the microstructure of the cured binder and consequently influences the fracture performance of CAEM. The microstructure of the cured binder is shown in Figure 19. It is evident that, at lower asphalt emulsion contents, the hydration products of the cement are clearly visible in the cured binder, where asphalt is segmented and encapsulated by these hydration products, resulting in discontinuous asphalt films. In this scenario, the binder forms a skeletal framework primarily composed of hydration products, with aggregates bonded mainly through these products. At this point, CAEM predominantly exhibits cement-based material characteristics, leading to a higher peak load but lower deformation capacity, thereby causing the specimen to fail via brittle fracture. With an increase in the asphalt emulsion content, the hydration products in the cured binder progressively diminish, and asphalt films gradually form a continuous structure. The asphalt films encapsulate the dispersed hydration products, which exist as discrete particulates. In this state, the binder forms a skeletal framework dominated by asphalt, with aggregates bonded primarily through asphalt. Consequently, CAEM predominantly exhibits asphalt-based material characteristics, resulting in a lower peak load but higher deformation capacity, causing the specimen to fail through ductile fracture.

### 4.5. Creep Compliance Analysis of CAEM

**Figure 20:**
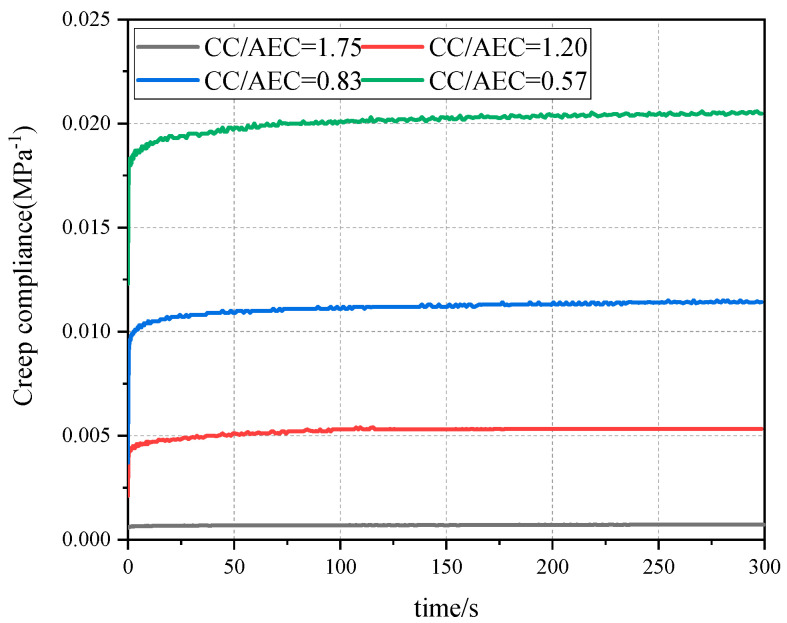
Creep compliance of CAEM.

Creep compliance is defined as the strain generated in a specimen under unit load, and it serves as a parameter for comparative analysis of material deformation capacity. The creep compliance of CAEM was obtained through creep tests, as shown in Figure 20. From the figure, it is evident that at identical loading durations, the creep compliance of CAEM increased with higher asphalt emulsion content. Furthermore, both the instantaneous elastic strain at t = 0 and the creep rate in the accelerated creep stage increased with an increase in asphalt content. When CC/AEC = 1.75 (AEC = 2.0%), the creep curve approximates a horizontal line, with the creep rate in the steady-state stage approaching zero, indicating that CAEM exhibited elastic behavior with negligible creep deformation. Conversely, at CC/AEC = 1.75 (AEC = 3.5%), the strain showed noticeable growth with prolonged loading duration, revealing that CAEM exhibited viscoelastic deformation characteristics and enhanced deformation capacity. The creep behavior of CAEM is intrinsically governed by its binder’s material composition and microstructure. Notably, CAEM exhibits cement-based material properties with negligible creep deformation at lower asphalt emulsion contents. In contrast, at higher asphalt emulsion contents, its behavior aligns with that of asphalt-based materials, demonstrating pronounced viscoelastic creep characteristics and enhanced deformation capacity. CAEM can undergo substantial deformation before failure, making it less prone to fracture failure. The increased asphalt emulsion content modifies the microstructure of the cured binder, thereby enhancing deformation capacity and improving the crack resistance of CAEM to a certain extent.

## 5. Conclusions

The binder of CAEM comprises cement and asphalt emulsion, two materials with divergent physicochemical properties. The asphalt emulsion content influences the material composition and microstructure of the cured binder. As the asphalt emulsion content increased, the skeletal framework of the binder transitioned from being dominated by cement hydration products to a network of asphalt films, thereby enhancing the viscoelastic deformation capacity of CAEM. This reorganization shifted the fracture mode from brittle to ductile failure mechanisms. The asphalt emulsion modified CAEM’s material performance, improving crack resistance to a certain extent.

Based on the material characteristics of CAEM, a comparative analysis of peak load and fracture energy was conducted. Fracture energy proved more appropriate for evaluating the fracture performance of CAEM. Peak load was correlated with the strength of the material, while the fracture performance of CAEM depended on both the strength and deformation capacity of the material. CAEM attained its maximum crack resistance when an optimal balance between strength and deformation capacity was achieved.

The influence of strength and deformation capacity on the fracture performance of CAEM is temperature-dependent, as dictated by its material characteristics. For the CAEM investigated in this study, crack resistance at lower temperatures was primarily governed by strength, whereas fracture behavior at elevated temperatures was dominated by deformation capacity. This dual dependency suggests that future research should focus on enhancing deformation capability at low temperatures and improving strength at high temperatures to optimize the crack resistance of CAEM.

This study establishes reference control parameter ranges for the mix design of CAEM. To achieve optimal crack resistance, the asphalt emulsion content should be maintained within 2.0~3.0%, while the binder content should be controlled below 6.0%.

Due to its intrinsically heterogeneous material characteristics, CAEM’s fracture behavior exhibited multifactorial dependencies. Among all the influencing factors, the material composition had the greatest impact on the fracture performance of CAEM. Beyond intrinsic compositional factors, external variables such as temperature, loading rate, and notch depth exerted significant influences on its fracture behavior, with coupling effects observed among these parameters. Therefore, future research should investigate the interactive effects of fracture-influencing factors in CAEM to advance mechanistic understanding and establish a scientific basis for optimizing its crack resistance.

## Figures and Tables

**Table 1:** Material parameters of asphalt emulsion.

Solid Content (%)	Evaporation Residue of Asphalt Emulsion		
	Softening Point (°C)	25 °C Penetration (0.1 mm)	15 °C Ductility (cm)
60.0	46.0	77	107

**Table 2:** Material parameters of cement.

Density (g/cm^3^)	Initial Setting Time (min)	Final Setting Time (min)	Flexural Strength (3d) (MPa)	Flexural Strength (28d) (MPa)	Compressive Strength (3d) (MPa)	Compressive Strength (28d) (MPa)
3.1	105	350	6.0	8.3	23.5	45.4

**Table 3:** Material parameters of coarse aggregate.

Aggregate Size (mm)	Bulk Volume Relative Density	Apparent Relative Density	Water Absorption (%)	Needle Flake Content (%)	Crushing Value (%)
15–20	2.752	2.784	0.26	6.2	9.6
10–15	2.767	2.788	0.56	9.4	8.2
5–10	2.759	2.779	0.80	7.6	8.2

**Table 4:** Material parameters of fine aggregate.

Aggregate Size (mm)	Apparent Relative Density	Mud Content (%)
3–5	2.711	2.1
0–3	2.686	2.7

**Table 5:** Material parameters of the mineral filler.

Density (g/cm^3^)	Water Content (%)	Percent Passing (%)			
		0.6 mm	0.3 mm	0.15 mm	0.075 mm
2.685	0.3	100	100	97.5	86.7

**Table 6:** Material proportions.

Specimen Number	CC (%)	AEC (%)	CC/AEC	BC (%)
AEC_2_CC_1_	2.0	2.0	1.00	4.0
AEC_3_CC_1_		2.5	0.80	4.5
AEC_4_CC_1_		3.0	0.67	5.0
AEC_5_CC_1_		3.5	0.57	5.5
AEC_1_CC_2_	2.5	1.5	1.67	4.0
AEC_2_CC_2_		2.0	1.25	4.5
AEC_3_CC_2_		2.5	1.00	5.0
AEC_4_CC_2_		3.0	0.83	5.5
AEC_5_CC_2_		3.5	0.71	6.0
AEC_1_CC_3_	3.0	1.5	2.00	4.5
AEC_2_CC_3_		2.0	1.50	5.0
AEC_3_CC_3_		2.5	1.20	5.5
AEC_4_CC_3_		3.0	1.00	6.0
AEC_1_CC_4_	3.5	1.5	2.33	5.0
AEC_2_CC_4_		2.0	1.75	5.5
AEC_3_CC_4_		2.5	1.40	6.0
AEC_5_CC_4_		3.5	1.00	7.0

**Table 7:** SCB test conditions.

Test Condition	T (°C)	LR (mm/min)	ND (mm)	Replicates
T_1_LS_1_ND_1_	20	1	10	3
T_1_LS_1_ND_2_	20	1	20	3
T_1_LS_1_ND_3_	20	1	30	3
T_1_LS_2_ND_2_	20	2	20	3
T_1_LS_3_ND_2_	20	3	20	3
T_2_LS_1_ND_1_	0	1	10	3
T_2_LS_1_ND_2_	0	1	20	3
T_2_LS_1_ND_3_	0	1	30	3
T_3_LS_1_ND_1_	−20	1	10	3
T_3_LS_1_ND_2_	−20	1	20	3
T_3_LS_1_ND_3_	−20	1	30	3

## Data Availability

The original contributions presented in the study are included in the article; further inquiries can be directed to the corresponding author.
